# SOCIAL ISOLATION AND RISK OF DEMENTIA: DO RACE, ETHNICITY, AND GENDER MATTER?

**DOI:** 10.1093/geroni/igad104.0902

**Published:** 2023-12-21

**Authors:** Zhiyong Lin, Hui (Cathy) Liu

**Affiliations:** University of Texas at San Antonio, San Antonio, Texas, United States; Purdue University, West Lafayette, Indiana, United States

## Abstract

Social isolation has emerged as a significant predictor of dementia risk in later life. However, our understanding of how this association may vary across race-ethnicity and gender remains limited. To address this gap, we used longitudinal data from the Health and Retirement Study (2000-2016) to examine the link between social isolation and dementia risk among older adults, separately for non-Hispanic Whites, non-Hispanic Blacks, and Hispanics. We also explored gender variations in this association within each racial-ethnic group. Our results indicate that social isolation increases the risk of dementia for both non-Hispanic Whites and Blacks, even after controlling for basic sociodemographic characteristics. However, social isolation does not appear to be a significant predictor of dementia risk among Hispanics, suggesting that cultural and social factors may play a key role in the association between social isolation and dementia risk across racial/ethnic groups. Further, we found few significant gender variations in the association between social isolation and dementia, except for a stronger effect of severe isolation among Black men compared to Black women. Economic resources and health-related factors were identified as significant mediators of the relationship between social isolation and dementia risk for both non-Hispanic Whites and Blacks. Our study highlights the importance of addressing social isolation as a potential risk factor for dementia among older adults, particularly for those in vulnerable groups. The findings underscore the need for targeted interventions to reduce social isolation and promote social engagement in diverse communities to mitigate dementia risk.

